# Dietary Curcumin Powder Supplementation Improves Growth Performance, Nutrient Digestibility, Nitrogen Utilization, Rumen Fermentation, and Plasma Antioxidant Activity in Meat Goats

**DOI:** 10.3390/ani16182952

**Published:** 2026-09-20

**Authors:** Pawinee Archa, Siwaporn Paengkoum, Nittaya Taethaisong, Pramote Paengkoum

**Affiliations:** 1 School of Animal Technology and Innovation, Institute of Agricultural Technology, Suranaree University of Technology, Muang, Nakhon Ratchasima 30000, Thailand; pawinee.a@sut.ac.th (P.A.); iszy.nittaya@gmail.com (N.T.); 2 Program in Agriculture, Faculty of Science and Technology, Nakhon Ratchasima Rajabhat University, Muang, Nakhon Ratchasima 30000, Thailand; siwaporn.p@nrru.ac.th

**Keywords:** curcumin powder, meat goats, growth performance, nutrient digestibility, nitrogen utilization, rumen fermentation, plasma antioxidant activity

## Abstract

This study evaluated the effects of dietary curcumin powder supplementation on growth performance, nutrient intake and digestibility, nitrogen utilization, rumen fermentation, blood urea nitrogen (BUN), and plasma antioxidant activity in meat goats. Twenty-four crossbred male goats (Thai native × Anglo-Nubian; initial body weight, 25.80 ± 2.06 kg) were assigned to four dietary treatments containing 0, 0.5, 1.0, or 1.5% curcumin powder in the concentrate. Dietary curcumin powder supplementation significantly affected final body weight and nutrient intake and produced dose-dependent responses in apparent nutrient digestibility and nitrogen utilization. In contrast, dietary treatment did not significantly affect the major rumen fermentation variables or BUN. Plasma ABTS and DPPH radical-scavenging activities showed significant linear responses to increasing curcumin supplementation at 2 and 4 h after feeding. Overall, these findings indicate that dietary curcumin powder supplementation was associated with greater final body weight and nutrient intake, dose-dependent changes in nutrient digestibility and nitrogen utilization, and increased plasma ABTS and DPPH radical-scavenging activities in meat goats, whereas the major rumen fermentation variables and BUN were not significantly affected by dietary treatment.

## Simple Summary

Natural feed additives are increasingly being explored as alternatives to conventional feed additives to improve animal productivity and health. Curcumin, the major bioactive curcuminoid in turmeric, has antioxidant, anti-inflammatory, and antimicrobial properties and may have potential as a natural feed additive for ruminants. This study found that dietary curcumin powder supplementation was associated with greater final body weight and nutrient intake, dose-dependent changes in nutrient digestibility and nitrogen utilization, and greater plasma ABTS and DPPH radical-scavenging activities after feeding. However, dietary curcumin powder supplementation did not significantly affect the major rumen fermentation variables measured.

## 1. Introduction

Goat production contributes substantially to food security and rural livelihoods, particularly in tropical and subtropical regions where goats are raised under diverse nutritional and environmental conditions. Meat goats are valued for their adaptability, reproductive efficiency, and ability to utilize fibrous feed resources. Nevertheless, growth performance and production efficiency in goats may be constrained by poor-quality diets, inefficient nutrient utilization, suboptimal rumen fermentation, and oxidative stress. An appropriate diet containing essential nutrients can significantly improve their growth, development, and reproductive health, resulting in better breeding performance, fewer health problems, and greater productivity [1,2]. These challenges have increased interest in natural feed additives that can improve feed utilization, animal health, and productive performance without relying on antibiotic growth promoters. The shift toward sustainable and safe livestock production has led to an increasing global focus on natural, green, and multifunctional feed additives to replace sub-therapeutic antibiotics [3]. Curcumin exhibits a broad spectrum of biological activities, including antioxidant, anti-inflammatory, antimicrobial, and immuno-modulatory properties [4,5]. More broadly, phytogenic feed additives contain biologically active compounds capable of interacting with the rumen microbial ecosystem and host metabolism, potentially affecting nutrient digestion, rumen fermentation, antioxidant defense, and productive performance. Evidence from ruminants indicates that supplementation with plant-derived polyphenolic compounds can improve dry matter intake, nutrient digestibility, average daily gain, serum antioxidant status, and ruminal propionate production while reducing lipid peroxidation [6]. However, responses vary according to the chemical characteristics of the bioactive compound, dietary composition, supplementation level, animal species, physiological stage, and duration of supplementation [6]. A key goal for improving goat health, productivity, and sustainability is the development of effective nutritional strategies. Such research not only responds to current agricultural challenges but also contributes to the development of more sustainable farming practices worldwide [7]. The development of effective feed supplements based on natural plant extracts is important for improving overall animal health and production efficiency [8,9]. Despite advances in research into these extracts and their potential benefits for goats, further research is still needed to fully understand the mechanisms of action of feed ingredients and their optimal doses to maximize their beneficial effects on animal health and productivity [7].

Curcumin is the major bioactive curcuminoid present in turmeric (*Curcuma longa* L.) and has attracted considerable attention as a potential natural feed additive because of its antioxidant, anti-inflammatory, antimicrobial, and metabolic regulatory properties. Its antioxidant activity is related partly to the phenolic hydroxyl and β-diketone moieties in its molecular structure, which contribute to free-radical scavenging and inhibition of oxidative chain reactions [10]. Oxidative stress can disrupt cellular redox homeostasis and may divert nutrients and metabolic resources away from processes associated with tissue growth and nutrient utilization. Consequently, improved antioxidant status may help maintain physiological conditions favorable for productive performance. Curcumin may contribute to this response through its radical-scavenging properties and regulation of antioxidant pathways. In addition to direct antioxidant activity, curcumin can modulate endogenous cellular antioxidant systems. In particular, activation of nuclear factor erythroid 2-related factor 2 (Nrf2)-dependent signaling promotes the expression of antioxidant-response genes and enzymes involved in cellular redox homeostasis, whereas suppression of nuclear factor-κB (NF-κB)-mediated signaling can attenuate inflammatory responses [11]. These complementary mechanisms may contribute to protection against oxidative and inflammatory damage associated with environmental, nutritional, and metabolic stress. In livestock, dietary phytochemicals with antioxidant activity have consequently attracted interest as nutritional strategies for maintaining redox homeostasis and productive performance. Supporting this concept, a meta-analysis of 36 studies in beef and dairy cattle demonstrated that dietary flavonoids increased serum superoxide dismutase (SOD), glutathione peroxidase (GSH-Px), and total antioxidant capacity (T-AOC), while decreasing malondialdehyde (MDA), an indicator of lipid peroxidation [6]. Although flavonoids and curcumin represent different subclasses of plant polyphenols, these findings provide broader evidence that dietary polyphenolic compounds can enhance antioxidant defense in ruminants. In addition to its systemic antioxidant effects, curcumin may influence animal performance through modulation of rumen fermentation and microbial activity. The rumen microbial ecosystem converts dietary carbohydrates and nitrogenous compounds into volatile fatty acids (VFAs) and microbial protein, which provide major sources of metabolizable energy and amino acids to the host animal. Consequently, changes in microbial populations, enzymatic activity, fermentation patterns, and microbial protein synthesis may affect nutrient utilization and animal growth. Direct evidence for these effects has been reported in growing lambs. Tian et al. [5] evaluated dietary curcumin at 0, 300, 600, and 900 mg/kg and observed improved average daily gain, altered ruminal fermentation, enhanced microbial protein synthesis, and increased serum antioxidant capacity. Curcumin supplementation also altered specific ruminal microbial populations, including increased *Prevotella ruminicola*, whereas methanogens, protozoa, and *Fibrobacter succinogenes* decreased with increasing curcumin supplementation [5]. The biological responses of ruminants to curcumin may depend on the level of dietary supplementation, and increasing inclusion does not necessarily result in proportionally greater beneficial effects. Excessive curcumin supplementation may adversely affect specific ruminal microorganisms, including fibrolytic bacteria such as *Fibrobacter succinogenes*, with potential consequences for fiber degradation and nutrient utilization. Therefore, identifying an appropriate dietary inclusion level is important for maximizing the potential benefits of curcumin while minimizing undesirable effects on the ruminal microbial ecosystem. Notably, excessive supplementation reduced the abundance of *Fibrobacter succinogenes*, indicating that high curcumin concentrations may inhibit important fibrolytic microorganisms and compromise the expected growth response [5].

The effects of curcumin on ruminal fermentation may be related partly to its selective antimicrobial activity and its interactions with microbial cell membranes, proteins, and metabolic enzymes. Through these actions, curcumin may alter microbial competition and fermentation pathways, potentially affecting ruminal ammonia nitrogen, VFA production, microbial protein synthesis, and the molar proportions of individual VFA. Changes in propionate production are of particular nutritional interest because propionate is the principal gluconeogenic VFA in ruminants and therefore contributes substantially to hepatic glucose production and energy metabolism. Broader evidence from ruminants supports the potential of dietary plant polyphenols to alter fermentation and nutrient utilization. A meta-analysis in beef and dairy cattle showed that dietary flavonoids increased dry matter digestibility and daily weight gain and increased the molar proportion of ruminal propionate [6]. Nevertheless, the effects of curcumin itself may vary among ruminant species and physiological conditions. Novakoski et al. [12] evaluated dietary curcumin in post-weaning dairy calves and observed enhanced antioxidant and anti-inflammatory responses, including increased catalase and SOD activities and reduced lipid peroxidation, but did not detect improvements in body weight or apparent nutrient digestibility [12]. These findings demonstrate that curcumin responses are not universally positive and emphasize the importance of species, dose, and diet specific evaluation [12].

Despite growing evidence supporting the biological activities of curcumin, research in ruminants remains relatively limited compared with that in monogastric species, and available studies have predominantly focused on sheep and cattle [5,12]. The biological responses of ruminants to curcumin may depend on the dietary inclusion level, and excessive supplementation may adversely affect specific ruminal microorganisms, including fibrolytic bacteria, potentially influencing nutrient utilization. Therefore, identifying an appropriate inclusion level is important for obtaining desirable responses while minimizing potential adverse effects. However, information on the responses of meat goats to graded levels of dietary curcumin remains limited, particularly when growth performance, nutrient utilization, rumen fermentation, and antioxidant activity are evaluated concurrently. This knowledge gap provides the rationale for evaluating multiple supplementation levels rather than a single curcumin dose. Therefore, the present study evaluated dietary curcumin powder at 0, 0.5, 1.0, and 1.5% of the concentrate diet to determine its effects on growth performance, nutrient intake and digestibility, nitrogen utilization, rumen fermentation, BUN, and plasma antioxidant activity in meat goats. We hypothesized that these responses would vary according to the level of dietary curcumin supplementation.

## 2. Materials and Methods

### 2.1. Experiment Location

The experimental protocol was approved by the Animal Care and Use Committee of Suranaree University of Technology (SUT 4/2558), Nakhon Ratchasima, Thailand. The study was conducted at the Suranaree University of Technology goat and sheep research farm in Nakhon Ratchasima, Thailand, in accordance with the recommendations of the National Research Council of Thailand on animal experimentation and the Animal Welfare Guidelines for Research (B-31/2562).

### 2.2. Animals, Treatments, and Experimental Design

The experiment was conducted at the Suranaree University of Technology (SUT) goat and sheep research farm in Nakhon Ratchasima, Thailand, from July to October 2025. The study area has a tropical savanna climate. During the experimental period, the mean ambient temperature was 33 °C, with a range of 31.0–33.8 °C, and the mean relative humidity was 80%. The goats were housed individually in pens measuring 1 × 2 m/goat. Twenty-four crossbred male goats (Thai native × Anglo-Nubian) with an initial body weight of approximately 25.80 ± 2.06 kg (mean ± SD) were used in a completely randomized design (CRD). The experiment lasted a total of 90 days, including the metabolic-cage adaptation and fecal and urine collection periods. The first 7 days were allocated to dietary adaptation, during which goats were adapted to their assigned experimental diets. Thereafter, all goats continued to receive their respective experimental diets throughout the remainder of the 90-day experimental period. During the final 10 days of the experiment, goats were transferred to individual metabolic cages. The first 3 days in the metabolic cages were used for adaptation, and were immediately followed by 7 consecutive days of total fecal and urine collection. Thus, the 3-day metabolic-cage adaptation and 7-day collection periods were included within, rather than added to, the 90-day experiment. The assigned experimental diets were continuously provided throughout these periods. Twenty-four goats were assigned to four dietary treatments, with six goats per treatment. Before treatment allocation, goats were ranked according to initial body weight and distributed among the four treatments to achieve comparable initial body weights across treatments. Within this allocation procedure, animals were randomly assigned to treatments, resulting in six goats per treatment. Each goat was housed and fed individually. Accordingly, the individual goat was considered the experimental unit (*n* = 6 per treatment) for all animal-level response variables. For rumen fermentation, blood urea nitrogen, and antioxidant variables measured at 0, 2, and 4 h relative to feeding, sampling time was treated as a repeated measure within the same goat. The ingredients and chemical composition of the concentrate diet with curcumin powder levels are shown in Table 1. The animals were housed in clean individual pens with free access to water. Before the experiment, all animals underwent a clinical health examination. The goats were fed diets at a roughage-to-concentrate ratio of 60:40. Curcumin powder was incorporated into the concentrate at 0, 0.5, 1.0, and 1.5% of concentrate DM for T1, T2, T3, and T4, respectively. Thus, the stated supplementation levels refer to the concentrate fraction and should not be interpreted as percentages of total dietary DM. The diets were offered in equal portions twice daily at 07:00 and 16:00 for ad libitum intake, allowing for approximately 10% refusal. The experimental diets were formulated to provide adequate nutrient supply for growing goats within the body-weight range used in the present study, according to the nutrient recommendations of NRC (2007) [13]. The ingredient composition and analyzed chemical composition of the experimental diets are presented in Table 1.

The curcumin used in the present study was commercially obtained from Wuxi Shiji Biotechnology Co., Ltd. (Wuxi, China) under the product name Curcumin, lot no. GB1886.76-2015. According to the manufacturer, the product contained ≥98% curcumin. Curcumin purity was analyzed by high-performance liquid chromatography (HPLC) following the method described by Marcon et al. [14], which verified a curcumin content of 97.72%. Other curcuminoids, including dimethoxycurcumin and bisdemethoxycurcumin, were detected at low concentrations (1.21% and 1.07%, respectively). The curcumin product was incorporated into the concentrate at inclusion levels of 0% (control), 0.5%, 1.0%, and 1.5% of concentrate DM and was thoroughly mixed to ensure uniform distribution before feeding.

### 2.3. Sample Collection

#### 2.3.1. Data Collection and Sampling Procedures

Individual feed intake was recorded daily throughout the 90-day experimental period. The amounts of feed offered and refusals were weighed daily for each goat, and dry matter intake (DMI) was calculated as the difference between the dry matter of feed offered and that of feed refused. Representative samples of the experimental diets and feed refusals were collected during the collection period and composited for each animal. The composite samples were dried in a forced-air oven at 65 °C for 72 h, ground to pass through a 1 mm screen using a Wiley mill, and stored in airtight containers at 4 °C until chemical analysis.

Dry matter intake relative to body weight was calculated individually for each goat using the mean body weight during the experimental period. Mean body weight was calculated as the average of initial and final body weights:

Mean BW (kg) = [Initial BW (kg) + Final BW (kg)]/2.

DMI as a percentage of body weight was calculated as:

DMI (% BW) = [DMI (kg/day)/mean BW (kg)] × 100.

Dry matter intake relative to metabolic body weight was calculated as:.

DMI (g/kg BW^0.75^) = DMI (g/day)/[mean BW (kg)]^0.75^.

Nutrient intake (g/day) = nutrient offered (g/day) − nutrient refused (g/day),

where Nutrient offered (g/day) = DM offered (g/day) × nutrient concentration in the offered diet (g/g DM), and Nutrient refused (g/day) = DM refused (g/day) × nutrient concentration in the refusals (g/g DM).

Accordingly, crude protein (CP), neutral detergent fiber (NDF), acid detergent fiber (ADF), and other nutrient intakes were calculated using their respective concentrations in the diet offered and refusals. Daily values were averaged for each individual goat over the experimental period and used for statistical analysis.

#### 2.3.2. Chemical Analyses

Samples were dried in an forced-air oven at 100 °C until they reached a constant weight to evaluate dry matter (DM) content before they were ground through a 1 mm screen using a Wiley hammer mill. Ash content was determined by combustion at 550 °C for 3 h in a muffle furnace. Neutral detergent fiber (NDF) was analyzed using a modified Van Soest method [15], with the addition of heat-stable amylase, while acid detergent fiber (ADF) was analyzed according to Van Soest et al. [15]. Hemicellulose content was calculated as the difference between NDF and ADF, while cellulose content was calculated as the difference between ADF and ADL. Nitrogen content was measured following AOAC [16] protocols, and crude protein (CP) content was calculated as N × 6.25.

#### 2.3.3. Fecal and Urine Collection

At the end of the feeding trial, goats were transferred to individual metabolic cages specifically designed to permit the separate collection of feces and urine. Before sample collection, the animals were allowed 3 days to acclimatize to the metabolic cages. This was followed by a 7-day collection period.

Feces and urine were collected separately from each goat on each of the seven consecutive collection days. Total daily fecal output was weighed and recorded, thoroughly mixed, and a representative subsample of approximately 100 g was collected. Daily fecal samples were dried in a forced-air oven at 65 °C for 72 h, ground through a 1 mm screen, and composited by animal before chemical analysis.

Urine was collected separately into plastic containers containing sulfuric acid to maintain the final urinary pH below 3 and thereby minimize nitrogen loss through ammonia volatilization. The total urinary output from each animal was measured daily, and approximately 100 mL of representative urine was collected daily. Urine samples were stored at −20 °C. At the end of the collection period, daily urine samples were pooled by animal for determination of urinary nitrogen concentration.

Total nitrogen concentrations in feed, refusals, feces, and urine were determined by the Kjeldahl method according to AOAC procedures. Nitrogen utilization was calculated individually for each goat using the following equations:

N intake (g/day) = feed DM intake (g/day) × N concentration of the consumed diet (g/g DM);

Fecal N (g/day) = total fecal DM output (g/day) × fecal N concentration (g/g DM);

Urinary N (g/day) = total urinary output (L/day) × urinary N concentration (g/L);

N absorption (g/day) = N intake − fecal N;

N balance (g/day) = N intake − fecal N − urinary N;

Fecal N excretion (% of N intake = (fecal N/N intake) × 100;

Urinary N excretion (% of N intake = (urinary N/N intake) × 100;

N balance (% of N intake) = (N balance/N intake) × 100.”

#### 2.3.4. Apparent Nutrient Digestibility and Nitrogen Balance

Apparent nutrient digestibility was estimated using acid-insoluble ash (AIA) as an internal marker according to Furuichi and Takahashi [17]. Representative dried and ground samples were treated with 4 N HCl and boiled for 30 min. The acid-insoluble residue was recovered by filtration and washed to remove residual acid and soluble material. The residue was then ashed at 650 °C for 6 h, cooled, and weighed. The resulting acid-insoluble ash concentration was expressed relative to the sample dry matter and used as an internal marker for the estimation of apparent nutrient digestibility. The apparent digestibility coefficient of each nutrient was calculated using the following equation:

Apparent nutritional digestibility (%) = 100 − [100 × (AIA diet/AIA feces) × (Nutrient feces/Nutrient diet)]

where AIA diet and AIA feces represent the concentrations of acid-insoluble ash in the diet and feces, respectively, and nutrient diet and nutrient feces represent the concentrations of the respective nutrients in the diet and feces.

#### 2.3.5. Growth Performance

Daily dry matter intake (DMI) was calculated throughout the study. Each goat was weighed in the morning after fasting on days 1 and 90 of the experiment. Growth performance was calculated using the following equations: body weight change (BWC, kg) = final body weight (kg) − initial body weight (kg); average daily gain (ADG, g/day) = body weight gain (kg) × 1000/number of experimental days.

#### 2.3.6. Rumen Fluid Sampling and Fermentation Characteristics

At the end of the feeding period, rumen fluid samples were collected from each goat at 0 h, immediately before the morning feeding, and at 2 and 4 h after feeding. A flexible stomach tube was introduced orally and advanced into the rumen.

Rumen fluid was obtained using a stomach tube connected to a suction pump. Approximately 100 mL of rumen fluid was collected from each goat at each sampling time. Rumen pH was measured immediately after collection using a calibrated glass-electrode pH meter. Immediately following, the samples were filtered through four layers of gauze and separated into centrifuge tubes, which were stored at −20 °C until analysis.

For determination of volatile fatty acids (VFAs), rumen fluid aliquots were acidified with 10% (*v*/*v*) H_2_SO_4_ and stored at −20 °C until analysis. Ruminal volatile fatty acids (VFAs) were analyzed using a gas chromatograph equipped with a flame ionization detector (GC-FID^®^; Varian Star 3600, Varian Inc., Palo Alto, CA, USA) and an autosampler (Varian 8200CX, Varian Inc., Palo Alto, CA, USA). A 1 µL aliquot of the extract was injected in split mode at 1:10. The carrier gas used was hydrogen at a constant pressure of 20 psi. The analytes (acetic, propionic, butyric, valeric, and isovaleric acids) were separated by a CP-WAX 52CB capillary column (60 m × 0.25 mm; 0.25 µm stationary phase thickness). The initial column temperature was set at 80 °C for 1 min and increasing to 120 °C at a rate of 8 °C min−1, then up to 230 °C by 15 °C min−1, where it remained for 1 min. Injector and detector temperatures were set at 250 °C. The validation of the method comprised the following parameters: selectivity, linearity, linear range, repeatability, precision, limit of detection (LOD) and limit of quantification (LOQ) for acetate, propionate, and butyrate. Acetate, propionate, and butyrate were identified by comparison with authentic standards and quantified using their respective calibration curves, with concentrations expressed as mmol/L. Total VFA concentration was calculated as the sum of the quantified individual VFAs.

#### 2.3.7. Blood Collection and Blood Urea Nitrogen

At the end of the feeding trial, blood samples were collected from the jugular vein at 0 h (immediately before morning feeding) and at 2 and 4 h after feeding. Blood samples were collected into appropriate anticoagulant-containing tubes and centrifuged at 2000× *g* for 15 min at 4 °C. The resulting plasma was collected and stored at −20 °C until analysis of blood urea nitrogen (BUN) and antioxidant activity.

#### 2.3.8. Determination of Plasma Antioxidant Activity

Plasma antioxidant activity was evaluated using the 2,2-diphenyl-1-picrylhydrazyl (DPPH) and 2,2′-azino-bis (3-ethylbenzothiazoline-6-sulfonic acid) (ABTS) radical-scavenging assays according to previously described methods [18,19], with modifications.

For determination of DPPH radical-scavenging activity, a 0.1 mM DPPH solution was freshly prepared in methanol and protected from light. A 20 µL plasma sample was mixed with 100 µL of the DPPH working solution and incubated in the dark at room temperature for 30 min. Absorbance was then measured at 517 nm using a Multiskan GO microplate spectrophotometer (Thermo Fisher Scientific) with the corresponding assay reagents (Shanghai Meilian Biotechnology Co., Ltd., Shanghai, China). A DPPH solution without plasma was used as the control.

DPPH radical-scavenging activity was calculated as follows:

DPPH scavenging activity (%) = [(A_control − A_sample)/A_control] × 100,

where A_control represents the absorbance of the control reaction and A_sample rep-resents the absorbance of the reaction containing the plasma sample.

For the ABTS assay, ABTS radical cations (ABTS•+) were generated by mixing a 7 mM ABTS stock solution with 2.45 mM potassium persulfate. The mixture was incubated in darkness at room temperature for 12–16 h before use. The resulting ABTS•+ solution was diluted with phosphate-buffered saline to obtain pH 7.4 and an absorbance of 0.70 ± 0.02 at 734 nm.

Plasma samples were mixed with the ABTS working solution and incubated for 6 min at room temperature. Absorbance was measured at 734 nm using the Multiskan GO spectrophotometer. Antioxidant activity was calculated as percentage inhibition using the following equation:

ABTS scavenging activity (%) = [(A_control − A_sample)/A_control] × 100.

#### 2.3.9. Statistical Analysis

Statistical analyses were performed using SAS version 9.4 (SAS Institute Inc., Cary, NC, USA). The individual goat was considered the experimental unit. Growth performance, nutrient intake, apparent nutrient digestibility, and nitrogen-balance variables measured once or summarized over the experimental period were analyzed using the GLM procedure according to a completely randomized design. The overall effect of dietary treatment was evaluated using the omnibus F-test. When the overall treatment effect was significant (*p* < 0.05), treatment least-squares means were compared using the Tukey–Kramer adjustment for multiple comparisons Steel and Torrie [20]. Different superscript letters indicate significant pairwise differences among treatment means at *p* < 0.05. Because dietary curcumin supplementation was applied at four equally spaced levels (0, 0.5, 1.0, and 1.5% of concentrate DM), orthogonal polynomial contrasts were used to evaluate linear, quadratic, and cubic dose–response relationships. The contrast coefficients were proportional to −3, −1, 1, and 3 for the linear effect; 1, −1, −1, and 1 for the quadratic effect; and −1, 3, −3, and 1 for the cubic effect. These contrasts were tested using CONTRAST statements in PROC GLM. Polynomial contrast *p*-values were interpreted independently of the overall treatment F-test and Tukey–Kramer pairwise comparisons.

Growth performance, feed intake, apparent nutrient digestibility, and nitrogen-balance variables measured once or summarized over the experimental period were analyzed using the General Linear Model (GLM) procedure. The statistical model was:

Y_i__j_ = μ + T_i_ + ε_i__j_,

where Y_i__j_ is the observed response, μ is the overall mean, T_i_ is the fixed effect of dietary treatment (i = 1, 2, 3, and 4), and ε_i__j_ is the residual error.

Rumen fermentation variables, BUN, and antioxidant variables measured repeatedly at 0, 2, and 4 h relative to feeding were analyzed using a repeated-measures mixed model with the MIXED procedure of SAS. The statistical model was:

Y_i__j__k_ = μ + T_i_ + H_k_ + (T × H)_i__k_ + A_j_(_i_) + ε_i__j__k_,

where Y_i__j__k_ is the observed response, μ is the overall mean, T_i_ is the fixed effect of dietary treatment, H_k_ is the fixed effect of sampling time (0, 2, and 4 h), (T × H)_i__k_ is the interaction between dietary treatment and sampling time, A_j_(_i_) is the random effect of animal nested within treatment, and ε_i__j__k_ is the residual error.

## 3. Results

### 3.1. Growth Performance and Nutrient Intakes

Dietary supplementation with curcumin powder significantly affected growth performance and nutrient intake (Table 2; *p* < 0.05). Final body weight, body weight gain, and DMI expressed as a percentage of body weight were greatest in goats receiving 1.5% curcumin powder in the concentrate and showed significant linear and quadratic responses (*p* < 0.05). DMI expressed relative to metabolic body weight also increased across treatments, with the greatest value observed in goats receiving 1.5% curcumin powder in the concentrate. Similarly, intakes of DM, CP, EE, NDF, and ADF were greatest in goats receiving 1.5% curcumin powder (*p* < 0.05).

### 3.2. Nutrient Digestibility

Apparent nutrient digestibility showed distinct dose–response patterns with increasing dietary curcumin powder supplementation (Table 3). Dry matter (DM) digestibility ranged from 66.2 to 77.0% and exhibited a significant quadratic response to increasing curcumin supplementation (*p* < 0.01), with the lowest value observed in goats fed concentrate containing 0.5% curcumin powder and progressively greater values in goats fed concentrate containing 1.0% curcumin powder and goats fed concentrate containing 1.5% curcumin powder. Similarly, crude protein (CP) digestibility showed a significant quadratic response (*p* < 0.01), decreasing from 65.4% in the control group to 55.2% in goats fed concentrate containing 0.5% curcumin powder before increasing to 65.2 and 69.8% in goats fed concentrate containing 1.0% curcumin powder and goats fed concentrate containing 1.5% curcumin powder, respectively. Pairwise comparisons indicated that CP digestibility was greatest in goats fed concentrate containing 1.5% curcumin powder and lowest in goats fed concentrate containing 0.5% curcumin powder (*p* < 0.05). By contrast, ether extract (EE), neutral detergent fiber (NDF), and acid detergent fiber (ADF) digestibility exhibited significant linear responses to increasing dietary curcumin powder supplementation (*p* < 0.01, *p* < 0.05, and *p* < 0.01, respectively). EE digestibility ranged from 52.6% in goats fed concentrate containing 0.5% curcumin powder to 71.9% in goats fed concentrate containing 1.5% curcumin powder, whereas ADF digestibility increased from 30.5% in goats fed concentrate containing 0.5% curcumin powder to 55.2% in goats fed concentrate containing 1.5% curcumin powder. NDF digestibility ranged from 69.9 to 78.3%, with the greatest numerical value observed in goats fed concentrate containing 1.5% curcumin powder. No significant cubic responses were detected for DM, CP, EE, NDF, or ADF digestibility (*p* > 0.05). Overall, the polynomial contrasts indicated nonlinear responses of DM and CP digestibility and predominantly linear responses of EE, NDF, and ADF digestibility to increasing curcumin supplementation.

### 3.3. Nitrogen Balance

Dietary curcumin powder supplementation affected several indices of nitrogen utilization (Table 4). Nitrogen intake increased from 43.6 g/d in the control group to 51.5 g/d in goats fed concentrate containing 1.5% curcumin powder and exhibited significant linear (*p* = 0.003), quadratic (*p* = 0.008), and cubic (*p* = 0.012) responses to increasing curcumin supplementation. Nitrogen absorption increased from 38.8 g/d in the control group to 47.4 g/d in goats fed concentrate containing 1.5% curcumin powder, while N balance increased from 36.9 to 46.0 g/d; both variables exhibited significant linear responses (*p* = 0.01). In contrast, urinary N (g/d) was not significantly affected by increasing curcumin supplementation, with no significant linear (*p* = 0.22), quadratic (*p* = 0.53), or cubic (*p* = 0.85) response, although the lowest numerical value was observed in goats fed concentrate containing 1.5% curcumin powder (1.4 g/d). Fecal N (g/d) showed no significant linear or quadratic responses (*p* = 0.10 for both), but a significant cubic response was detected (*p* = 0.01).

### 3.4. Rumen Fermentation Parameter

Dietary curcumin powder supplementation had no significant effect on the major rumen fermentation characteristics, including total volatile fatty acids (VFAs), acetate, propionate, butyrate, the acetate-to-propionate ratio, and ruminal pH (*p* > 0.05) are presented in Table 5. Similarly, no significant treatment × time interactions were detected for these variables, indicating that the responses to curcumin supplementation were generally consistent across the sampling times. A significant sampling-time effect was observed for total VFA and acetate (*p* = 0.05), indicating temporal changes during the postprandial period rather than an effect of dietary treatment. Although goats supplemented with 1.0% curcumin powder showed a numerically greater total VFA concentration at 2 h (50.5 mmol/L), this increase was not statistically significant compared with the other treatments. Propionate, butyrate, and the acetate-to-propionate ratio were not significantly influenced by dietary treatment, sampling time, or their interaction (*p* > 0.05). Ruminal pH also remained stable across treatments and sampling times, ranging from approximately 6.9 to 7.5, indicating that supplementation with curcumin powder up to 1.5% of concentrate did not negatively affect the ruminal environment.

Blood urea nitrogen (BUN) concentrations are presented in Table 6. No significant effects of dietary treatment (*p* > 0.05), sampling time (*p* > 0.05), or the treatment × time interaction (*p* > 0.05) were observed for BUN concentrations. BUN values ranged from 12.7 to 15.0 mg/dL across treatments and sampling times, indicating that dietary curcumin powder supplementation did not markedly alter circulating BUN concentrations during the post-feeding period.

### 3.5. Plasma Antioxidant Activity

Plasma ABTS and DPPH radical-scavenging activities are presented in Table 7. At 0 h before feeding, ABTS activity was similar among treatments, ranging from 29.3% to 30.8%, and no significant differences among dietary treatments were observed. At 2 h after feeding, ABTS activity was greater in curcumin-supplemented goats than in the control group, increasing from 47.2% in T1 to 55.0%, 55.3%, and 57.9% in T2, T3, and T4, respectively. The greatest ABTS activity was observed in T4. A similar response was observed at 4 h, when ABTS activity increased from 48.4% in the control group to 79.2%, 79.8%, and 81.0% in T2, T3, and T4, respectively, with T4 showing the greatest value.

DPPH radical-scavenging activity also showed no appreciable differences among treatments at 0 h, with values ranging from 30.1% to 30.3%. In contrast, marked treatment differences were observed after feeding. At 2 h, DPPH activity increased from 34.6% in T1 to 68.9%, 69.5%, and 70.1% in T2, T3, and T4, respectively. At 4 h, DPPH activity increased progressively from 50.5% in T1 to 75.1%, 89.4%, and 93.5% in T2, T3, and T4, respectively. Overall, these results indicate that dietary curcumin supplementation was associated with greater plasma ABTS and DPPH radical-scavenging activities after feeding, with the highest values generally observed in goats receiving 1.5% curcumin powder. 

## 4. Discussion

Dietary curcumin powder supplementation was associated with greater final body weight, body weight gain, and nutrient intake in meat goats, with the greatest values generally observed in goats receiving 1.5% curcumin powder in the concentrate. These responses occurred together with changes in apparent nutrient digestibility and nitrogen utilization. However, the present study did not directly measure the mechanisms responsible for the growth response. Improvements in apparent nutrient digestibility and nitrogen utilization may have increased nutrient availability, but tissue nitrogen deposition and microbial protein synthesis were not measured. Likewise, although plasma ABTS and DPPH radical-scavenging activities were greater after feeding in curcumin-supplemented goats, these assays reflect radical-scavenging activity and do not directly demonstrate changes in endogenous antioxidant enzymes or oxidative damage. Previous studies in growing ruminants have reported positive effects of dietary curcumin on growth performance [21]. Tian et al. [5] reported improved average daily gain in growing lambs supplemented with curcumin, whereas Marcon et al. [14] observed greater average daily gain in lambs fed curcumin without an increase in feed intake. Glombowsky et al. [22] also reported greater weight gain in calves supplemented with curcumin. Collectively, these studies support the possibility that curcumin can influence productive performance in growing ruminants, although responses may depend on species, diet, dose, and experimental conditions.

Dietary curcumin powder supplementation affected apparent nutrient digestibility in meat goats, although the magnitude and pattern of the response differed among nutrient fractions. DM and CP digestibility exhibited significant quadratic responses, whereas EE, NDF, and ADF digestibility showed predominantly linear responses to increasing curcumin supplementation. The responses were characterized by an initial decline at 0.5% supplementation followed by recovery at 1.0% and generally greater digestibility at 1.5%. These patterns indicate that the effects of curcumin on nutrient digestibility were dose dependent rather than uniformly increasing across all supplementation levels. Previous studies provide some support for turmeric-derived bioactive compounds affecting nutrient utilization. Karhale et al. [23] reported that increasing turmeric powder supplementation in growing goats increased the apparent digestibility of several nutrients. Tian et al. [5] also reported effects of curcumin on ruminal microbial protein synthesis in growing lambs. Nevertheless, microbial protein synthesis and specific ruminal microbial populations were not measured in the present study; therefore, any microbial mechanism underlying the observed digestibility responses remains hypothetical.

Nitrogen balance is an important indicator of protein utilization in growing ruminants because it reflects the partitioning of dietary nitrogen among fecal and urinary losses and retained nitrogen. In the present study, dietary curcumin supplementation was associated with changes in several measured indices of nitrogen utilization, including greater nitrogen intake, nitrogen absorption, and nitrogen balance at higher supplementation levels. These responses should be interpreted specifically as changes in the measured nitrogen-balance variables. Tissue nitrogen deposition and microbial protein synthesis were not directly determined; therefore, the present findings do not establish that the additional dietary nitrogen was incorporated into productive tissue or microbial biomass. The greater nitrogen intake at the higher supplementation levels may also partly reflect greater feed and CP intake. Tian et al. [5] reported that dietary curcumin altered ruminal nitrogen metabolism and increased microbial protein synthesis in growing lambs, while Bokharaeian et al. [24] reported dose-dependent effects of curcumin nano-micelles on nitrogen metabolism and nutrient digestibility in fattening lambs. These studies provide possible explanations for the responses observed here, but direct measurements of microbial protein synthesis and tissue nitrogen deposition would be required to establish the underlying mechanisms in meat goats.

Volatile fatty acids (VFAs) are major end products of ruminal carbohydrate fermentation and provide an important source of metabolizable energy to ruminants. In the present study, however, dietary curcumin powder supplementation did not significantly affect total VFA concentration, acetate, propionate, butyrate, the acetate-to-propionate ratio, or ruminal pH. Moreover, no significant treatment × time interactions were detected for these variables. These findings indicate that, under the conditions of the present experiment, supplementation with curcumin powder at up to 1.5% of the concentrate did not produce a statistically detectable effect on the measured rumen fermentation characteristics. A sampling-time effect was observed for total VFA and acetate concentrations, indicating that these variables changed during the post-feeding period independently of dietary treatment. Such temporal variation is consistent with changes in ruminal fermentation following feed consumption; however, because no significant treatment × time interaction was detected, the post-feeding changes cannot be attributed specifically to curcumin supplementation. Although goats receiving 1.0% curcumin powder showed a numerically greater total VFA concentration at 2 h after feeding, this difference was not statistically significant and should therefore not be interpreted as evidence that curcumin enhanced ruminal fermentation. These fermentation responses may consequently contribute to the improvements in nutrient digestibility, nitrogen utilization, and growth performance observed in the present study. The effects of curcumin on ruminal VFA concentration are likely related to its antimicrobial properties. Unlike conventional antimicrobial compounds that may broadly suppress microbial fermentation, phytogenic compounds such as curcumin may selectively inhibit or stimulate specific groups of rumen microorganisms. This selective activity can modify the pathways through which dietary carbohydrates are fermented and consequently alter both total VFA concentration and the relative proportions of acetate, propionate, butyrate. Vitor et al. [14] reported that dietary curcumin changed the activity of enzymes involved in adenosine triphosphate (ATP) metabolism, thus developing weight gain in lambs. The increase in total VFA concentration after feeding may be related to enhanced degradation of dietary organic matter and structural carbohydrates. Volatile fatty acids are produced primarily through microbial fermentation of carbohydrates and supply a major proportion of metabolizable energy to ruminants. Therefore, greater total VFA concentration can be interpreted as evidence of increased microbial fermentation of dietary substrates, provided that ruminal absorption and passage rates are considered. This interpretation is consistent with the nutrient digestibility responses observed in the present study, particularly the greater DM, NDF, and ADF digestibility at the higher levels of curcumin supplementation.

Ruminal pH was not significantly affected by dietary curcumin supplementation. The observed values indicate that the ruminal environment remained relatively stable across treatments and sampling times. Because fibrolytic microorganisms are sensitive to low ruminal pH, maintenance of pH within the observed range would be compatible with conditions that support fiber digestion. However, the present study did not measure fibrolytic microbial abundance or activity, and the greater NDF and ADF digestibility observed at higher curcumin supplementation levels cannot be attributed directly to changes in these microorganisms. Overall, the repeated-measures analysis provides no evidence that curcumin supplementation altered the measured rumen fermentation variables, despite the observed changes in nutrient digestibility, nitrogen utilization, and growth performance.

Dietary curcumin powder supplementation did not significantly affect BUN concentrations, and no significant effects of sampling time or treatment × time interaction were detected. Thus, the present findings indicate that circulating BUN concentrations were not detectably altered by curcumin supplementation during the sampling period. These findings indicate that dietary curcumin did not substantially disturb systemic urea homeostasis in meat goats. Curcumin may influence this process through modulation of ruminal microbial activity. These findings suggest that an appropriate concentration of curcumin may improve the incorporation of available ruminal nitrogen into microbial biomass. This relationship provides a mechanistic connection with the improved growth performance observed in the present experiment. Supplementation with curcumin powder at 1.5% concentration in goats resulted in the greatest final live weight and daily weight gain and simultaneously led to greater CP intake, CP digestibility, nitrogen absorption, and nitrogen retention without an increase in BUN. These coordinated responses suggest that the additional dietary nitrogen was effectively utilized for productive tissue deposition. Therefore, the present findings indicate only that circulating BUN concentrations were not detectably altered by curcumin supplementation during the sampling period.

The potential antioxidant response is particularly relevant when considered together with the growth performance observed in the present study. Curcumin-supplemented goats, particularly those receiving higher supplementation levels, exhibited greater final live weight and daily weight gain. Dietary curcumin supplementation was associated with greater plasma ABTS and DPPH radical-scavenging activities after feeding, particularly at the higher supplementation levels. These findings indicate greater radical-scavenging activity in the plasma samples under the conditions of the ABTS and DPPH assays. Indeed, previous studies have noted that the antioxidants present in curcumin extracts enhance natural defense mechanisms, leading to a reduction in oxidative damage to lipids [25]. Previous studies have proposed that curcumin may influence oxidative status through its radical-scavenging properties and potential modulation of endogenous antioxidant systems. However, these mechanisms were not directly evaluated in the present study because ROS/RNS, antioxidant enzyme activities, endogenous antioxidant concentrations, and biomarkers of lipid peroxidation were not measured by the bioactive components present in curcumin; these are thought to enhance the defense against oxidative stress by increasing antioxidant enzyme activity and boosting endogenous antioxidant levels. This reduction in oxidative lipid damage contributes to an overall improvement in oxidative balance.

## 5. Conclusions

Among the dietary curcumin powder supplementation levels evaluated in the present study (0, 0.5, 1.0, and 1.5% of concentrate DM), the 1.5% level was associated with the greatest responses in several measured variables, including final body weight, average daily gain, nutrient intake, selected nutrient digestibility and nitrogen-utilization indices, and post-feeding plasma ABTS and DPPH radical-scavenging activities. In contrast, the major rumen fermentation variables and BUN were not significantly affected by dietary treatment. Because 1.5% was the highest supplementation level evaluated, the present study does not establish this level as the optimal inclusion rate. Further dose–response studies are required to determine the optimal dietary inclusion level and to confirm the practical applicability, long-term efficacy, and safety of curcumin powder supplementation in meat goats.

## Figures and Tables

**Table 1:** Ingredients and chemical composition of experimental diets.

Ingredients	Treatments			
	T1	T2	T3	T4
Soybean meal	31.0	31.0	31.0	31.0
Cornmeal	23.0	23.0	23.0	23.0
Cassava	21.5	21.0	20.5	20.0
Rough	23.0	23.0	23.0	23.0
Curcumin Powder	0.0	0.5	1.0	1.5
Molasses	1.0	1.0	1.0	1.0
Urea	0.3	0.3	0.3	0.3
Mineral premix	0.2	0.2	0.2	0.2
Total (kg/100 kg)	100	100	100	100
Composition, % of DM				
DM	88.0	87.0	87.1	87.1
Ash	7.9	6.0	6.9	6.5
CP	18.4	18.5	18.5	18.5
EE	2.8	2.8	2.8	2.8
NDF	54.0	54.1	54.5	54.8
ADF	30.6	31.1	31.6	31.9
TDN	38.0	37.7	37.3	37.0
Antioxidant activity (%)				
ABTS	0.0	50.3	55.7	63.7
DPPH	0.0	57.6	64.0	67.1

T1: control group; T2: supplemented with curcumin powder at 0.5% in a concentrate; T3: supplemented with curcumin powder at 1% in a concentrate; T4: supplemented with curcumin powder at 1.5% in a concentrate; DM: dry matter; CP: crude protein; EE: ether extract; NDF: neutral detergent fiber; ADF: acid detergent fiber; TDN: total digestible nutrients; ABTS: (2,2′-azinobis-(3-ethylbenzothiazoline-6-sulfonic acid); DPPH: (2,2-diphenyl-1-picrylhydrazyl).

**Table 2:** Effect of dietary curcumin powder supplementation on growth performance and nutrient intake in meat goat.

Parameter	Treatments				SEM	*p*-Value		
	T1	T2	T3	T4		Linear	Quadratic	Cubic
Initial weight, kg	25.8	25.7	25.0	25.0	2.47	0.60	0.51	0.55
Final weight, kg	29.5 ^d^	32.2 ^c^	33.1 ^b^	35.3 ^a^	0.67	0.01	0.01	0.41
body-weight gain, kg	3.7 ^d^	6.5 ^c^	8.1 ^b^	10.3 ^a^	0.71	0.01	0.01	0.23
ADG, g/d	41.1 ^d^	72.2 ^c^	90.0 ^b^	114.4 ^a^	0.71	0.01	0.01	0.23
% BW, kg	6.61 ^d^	6.68 ^cd^	6.87 ^bc^	7.16 ^a^	0.86	0.01	0.01	0.34
DMI, g/kg BW^0.75^	151.6 ^cd^	155.0 ^d^	159.4 ^bc^	167.8 ^a^	0.92	0.52	0.01	0.25
**Nutrient intake, g/d**								
DM	1827.8 ^d^	1934.8 ^c^	1994.3 ^b^	2159.5 ^a^	46.90	0.01	0.71	0.67
CP	186.7 ^d^	193.5 ^c^	199.1 ^b^	212.5 ^a^	3.57	0.01	0.58	0.73
EE	36.9 ^d^	38.3 ^c^	39.7 ^b^	42.9 ^a^	0.83	0.01	0.51	0.75
NDF	1221.6 ^d^	1277.2 ^c^	1325.4 ^b^	1437.8 ^a^	29.95	0.01	0.57	0.73
ADF	591.0 ^d^	620.2 ^c^	643.9 ^b^	701.5 ^a^	15.41	0.01	0.58	0.73

T1: control group; T2: supplemented with curcumin powder at 0.5% in a concentrate; T3: supplemented with curcumin powder at 1% in a concentrate; T4: supplemented with curcumin powder at 1.5% in a concentrate; SEM: standard error of mean; DM: dry matter; CP: crude protein; EE: ether extract; NDF: neutral detergent fiber; ADF: acid detergent fiber; a b c d in the same row indicates that there is a statistically significant difference (*p* < 0.05).

**Table 3:** Effect of dietary curcumin powder supplementation on nutrient digestibility in meat goats.

Parameter	Treatments				SEM	*p*-Value		
	T1	T2	T3	T4		Linear	Quadratic	Cubic
**Digestibility, %**								
DM	69.0	66.2	73.5	77.0	2.02	0.47	0.01	0.88
CP	65.4 ^b^	55.2 ^c^	65.15 ^b^	69.8 ^a^	2.55	0.69	0.01	0.97
EE	59.9 ^c^	52.6 ^d^	65.2 ^b^	71.9 ^a^	1.75	0.01	0.61	0.29
NDF	74.1 ^c^	69.9 ^d^	75.2 ^b^	78.3 ^a^	0.99	0.05	0.40	0.15
ADF	39.7 ^c^	30.5 ^d^	46.3 ^b^	55.2 ^a^	2.54	0.01	0.45	0.21

T1: control group; T2: supplemented with curcumin powder at 0.5% in a concentrate; T3: supplemented with curcumin powder at 1% in a concentrate; T4: supplemented with curcumin powder at 1.5% in a concentrate; SEM: standard error of mean; a b c d in the same row indicates that there is a statistically significant difference (*p* < 0.05).

**Table 4:** Effect of dietary curcumin powder supplementation on nitrogen balance in meat goats.

Item	Treatments				SEM	*p*-Value		
	T1	T2	T3	T4		Linear	Quadratic	Cubic
**Nitrogen balance**								
N intake (g/d)	43.6 ^d^	46.1 ^c^	47.6 ^bc^	51.5 ^a^	1.12	0.003	0.008	0.012
Fecal N (g/d)	4.7	4.6	4.4	4.0	1.03	0.10	0.10	0.01
Urinary N (g/d)	1.8	1.7	1.8	1.4	0.22	0.22	0.53	0.85
N absorption (g/d)	38.8	41.5	43.1	47.4	2.95	0.01	0.10	0.10
N balance (g/d)	36.9	39.7	41.2	46.0	3.92	0.01	0.10	0.10
Fecal N excretion (%)	10.78	9.98	9.24	7.77	0.46	0.46	0.05	0.15
Urine N excretion (%)	4.13 ^a^	3.69 ^c^	3.78 ^b^	2.72 ^d^	0.59	0.047	0.052	0.054

T1: control group; T2: supplemented with curcumin powder at 0.5% in a concentrate; T3: supplemented with curcumin powder at 1% in a concentrate; T4: supplemented with curcumin powder at 1.5% in a concentrate; SEM: standard error of mean; a b c d in the same row indicates that there is a statistically significant difference (*p* < 0.05).

**Table 5:** Effect of dietary curcumin powder supplementation on volatile fatty acids in meat goats.

Parameter	Treatments				SEM	*p*-Value		
	T1	T2	T3	T4		Treatment	Time	Treatment × Time
**Total VFA (mmol/L)**								
0 h	33.2	33.9	34.8	30.6	2.52	0.89	0.63	0.14
2 h	33.6	34.7	50.5	42.9	4.62	0.33	0.05	0.38
4 h	31.0	33.2	35.5	38.7	2.12	0.36	0.05	0.70
**Acetate** (**%Molar**)								
0 h	70.4	70.7	67.3	69.4	1.12	0.62	0.64	0.38
2 h	66.9	62.4	64.1	65.6	1.05	0.74	0.05	0.81
4 h	68.3	61.4	66.5	66.8	1.23	0.97	0.05	0.12
**Propionate** (**%Molar**)								
0 h	21.4	20.6	20.4	21.9	0.44	0.74	0.24	0.18
2 h	23.1	25.8	24.1	24.8	0.48	0.41	0.28	0.13
4 h	23.2	22.5	23.5	23.7	0.56	0.68	0.73	0.65
**Butyrate** (**%Molar**)								
0 h	8.0	8.58	12.1	8.1	1.18	0.72	0.37	0.34
2 h	9.9	11.7	11.6	9.5	2.90	0.45	0.21	0.99
4 h	8.3	15.9	9.8	9.4	1.62	0.85	0.23	0.19
**Acetate/Propionate**								
0 h	3.3	3.4	3.2	3.2	0.35	0.60	0.65	0.66
2 h	2.9	2.4	2.6	2.6	0.36	0.53	0.21	0.23
4 h	2.9	2.7	2.8	2.8	0.05	0.56	0.50	0.53
**Ruminal pH**								
0 h	7.3	7.4	7.1	7.5	0.12	0.82	0.55	0.46
2 h	7.0	7.3	7.3	6.9	0.12	0.78	0.23	0.99
4 h	7.1	7.3	7.3	7.3	0.12	0.65	0.69	0.81

T1: control group; T2: supplemented with curcumin powder at 0.5% in a concentrate; T3: supplemented with curcumin powder at 1% in a concentrate; T4: supplemented with curcumin powder at 1.5% in a concentrate; SEM: standard error of mean.

**Table 6:** Effect of dietary curcumin powder supplementation on blood urea nitrogen in meat goats.

Parameter	Treatments				SEM	*p*-Value		
	T1	T2	T3	T4		Treatment	Time	Treatment × Time
**BUN, mg/dL**								
0 h	14.0	14.2	13.5	14.0	0.19	0.68	0.76	0.23
2 h	15.0	14.2	13.7	14.0	0.35	0.32	0.52	0.88
4 h	14.7	12.7	14.2	14.0	0.38	0.82	0.26	0.13

T1: control group; T2: supplemented with curcumin powder at 0.5% in a concentrate; T3: supplemented with curcumin powder at 1% in a concentrate; T4: supplemented with curcumin powder at 1.5% in a concentrate; SEM: standard error of mean; BUN: blood urea nitrogen.

**Table 7:** Effect of dietary curcumin powder supplementation on plasma antioxidant activity in meat goats.

Parameter	Treatments				SEM	*p*-Value		
	T1	T2	T3	T4		Treatment	Time	Treatment × Time
**Antioxidant radical-scavenging activity (%)**								
**ABTS**, **(%)**								
0 h	29.8	29.8	30.8	29.3	0.65	0.16	0.35	0.27
2 h	47.2 ^d^	55.0 ^c^	55.3 ^bc^	57.9 ^a^	1.24	0.01	0.11	0.12
4 h	48.4 ^d^	79.2 ^c^	79.8 ^bc^	81.0 ^a^	3.60	0.01	0.11	0.12
**DPPH**, (%)								
0 h	30.1	30.3	30.2	30.2	1.27	0.35	0.83	0.94
2 h	34.6 ^d^	68.9 ^c^	69.5 ^b^	70.1 ^a^	3.90	0.01	0.33	0.44
4 h	50.5 ^d^	75.1 ^c^	89.4 ^b^	93.5 ^a^	4.38	0.01	0.53	0.51

T1: control group; T2: supplemented with curcumin powder at 0.5% in a concentrate; T3: supplemented with curcumin powder at 1% in a concentrate; T4: supplemented with curcumin powder at 1.5% in a concentrate; SEM: standard error of mean; ABTS: (2,2′-azinobis-(3-ethylbenzothiazoline-6-sulfonic acid); DPPH: (2,2-diphenyl-1-picrylhydrazyl); a b c d in the same row indicates that there is a statistically significant difference (*p* < 0.05).

## Data Availability

The datasets generated during the current study are available from the corresponding author on reasonable request.
